# Ocular toxicity due to Trametinib and Dabrafenib

**DOI:** 10.1186/s12886-017-0541-0

**Published:** 2017-08-17

**Authors:** Stephanie Sarny, Michael Neumayer, Julian Kofler, Yosuf El-Shabrawi

**Affiliations:** 1 General Hospital Klagenfurt, Department of Opthalmology, Feschnigstraße 11, A-9020 Klagenfurt, Austria; 2 General Hospital Klagenfurt, Department of Dermatology, Feschnigstraße 11, A-9020 Klagenfurt, Austria

**Keywords:** Trametinib, Dabrafenib, Ocular toxicity, Neuroretinal detachment, Uveitis anterior

## Abstract

**Background:**

To report a case of uveitis and neuroretinal detachment in a patient treated with Trametinib and Dabrafenib due to metastatic cutaneous melanoma stage IV.

**Case presentation:**

We evaluated slit lamp examination, fundoscopy, optical coherence tomography, fluorescein and indocyanine green angiography in a 66 years old man suffering visual loss. Fundoscopy showed serous neuroretinal detachment of the fovea accompanied with white spots surrounding the fovea in both eyes. Although therapy with Trametinib and Dabrafenib was stopped uveitis anterior was seen 2 weeks later. After a year, the therapy was started again and the serous neuroretinal detachment appeared once more, however without inflammatory reaction of the anterior chamber.

**Conclusion:**

Patients treated with Trametinib and Dabrafenib should undergo consecutive eye examinations from the beginning of the therapy.

## Background

Melanoma is the most lethal skin cancer. In recent years, new chemotherapeutics were developed to prolong progression free survival time. However, more and more authors report severe ocular side effects. The signal pathway, called mitogen-activated protein kinase (MAPK) pathway, plays a key role in the development of malignant melanoma. In about half of the patients a mutation in BRAF gene is detected. This mutation induces a transformation in the MAPK pathway and leads to a transcription of genes responsible for the survival of tumour cells.

Trametinib is a mitogen-activated protein kinase (MEK) inhibitor which is applied in cutaneous melanoma with a mutation in the gene BRAF V600E or V600 K. Of the melanomas 80–90% show a mutation in BRAF V600E and 10–20% in BRAF V600 K [1]. Trametinib is applied as single agent or in combination with Dabrafenib [2]. Dabrafenib is a BRAF-inhibitor which inhibits BRAF V600E kinase selectively and should reduce the proliferation of malignant tumour cells. A phase 3 clinical study with more than 700 patients was induced and showed a significantly prolonged progression-free survival time for the combination of Trametinib and Dabrafenib compared to the standard chemotherapeutic [3].

Recently authors report of ocular complications under therapy like central serous-like chorioretinopathy, uveitis or retinal vein occlusion [4, 5]. We report of a patient receiving Trametinib and Dabrafenib and developing both uveitis and bilateral central serous chorioretinopathy.

## Case presentation

A male patient aged 66 years with metastatic cutaneous melanoma stage IV of the back presented with visual loss on both eyes, painful eyes and foreign body sensation. No prior medication, illnesses or allergies were known. Chemotherapy was started with Dabrafenib 75 mg four times a day since 5 months and Trametinib 2 mg once a day since 3 months.

**Fig. 1:**
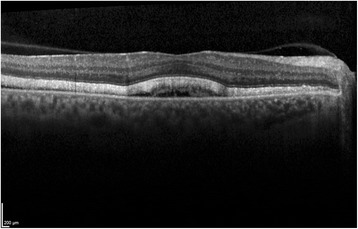
Optical coherence tomography of the right eye at first examination under therapy with Dabrafenib and Trametinib

**Fig. 2:**
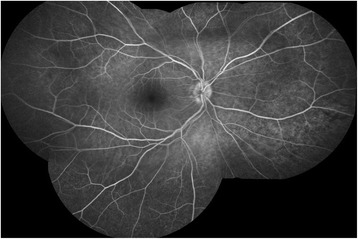
Fluorescein angiography of the right eye at first examination under therapy with Dabrafenib and Trametinib

**Fig. 3:**
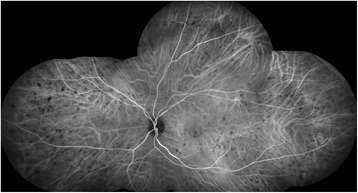
Indocyanine green angiography of the right eye at first examination under therapy with Dabrafenib and Trametinib

At first clinical visit visual acuity was 20/25 on both eyes. Slit lamp examination only revealed a dry eye, but funduscopy showed a bilateral serous neuroretinal detachment of the fovea accompanied with white spots surrounding the fovea. Optical coherence tomography presented a central retinal detachment on both eyes (Fig. 1). Fluorescein and indocyanine green angiography showed hypofluorescein spots without optic disc leakage (Figs. 2 and 3). The therapy with Trametinib and Dabrafenib was stopped immediately after diagnosis. We induced a uveitis work-up, including blood test and computer tomography, which were negative.

Although therapy was stopped, the patient presented again after 2 weeks with a visual loss. Visual acuity was then 20/40 on the right eye and 20/50 on the left eye. Examination also showed uveitis of the anterior segment with anterior chamber cells 1–2+ and partial synechiae of the left pupil, vitritis was not detected. Therefore, local therapy was started with topical corticosteroid treatment six times a day and mydriatic agents once a day. Within a week good response was obtained and uveitis symptoms and neuroretinal detachment reduced. Therefore, Dabrafenib was again started and after another week uveitis anterior became worse again on the left eye and appeared also on the right eye. Dabrafenib was completely stopped and a Triamcinolonacetonid parabulbous injection at the left eye was applied.

After 2 months without any therapy Nivolumab, a monoclonal antibody, was administered [6]. It binds at the receptor PD-1 of active T-cells and leads to an increased activity of T-cells. Visual acuity was 20/30 on both eyes and eye examination showed no uveitis or serous neuroretinal detachment.

After half a year the therapy with Dabrafenib and Trametinib was started again. The patient presented once more with a vision loss of 20/200 and a visual acuity of 20/40 on both eyes. Serous neuroretinal detachment was detected, however without any inflammatory process of the anterior segment.

After 1 year, clinical examination showed neuroretinal detachment only on the right eye and no signs of uveitis.

## Conclusions

Recently a few reports introduced the ocular side effects of MEK-inhibitors. Both Dabrafenib and Trametinib inhibit the MAPK pathway and are applied in the treatment of metastatic cutaneous melanoma. They prolong the progression free survival time. Reported ocular side effects are retinal vein occlusion with an incidence of <1.5% in Trametinib, chorioretinopathy with an incidence of up to 2% in both Trametinib and Dabrafenib and uveitis with unknown incidence [7]. Further agents for patients with malignant melanoma have been described. The BRAF-inhibitor Vemurafenib, usually in combination with the MEK-inhibitor cobimetinib, was associated with both uveitis in 4% [8] and with serous retinopathy in even 26% [9].

Our patient showed both chorioretinopathy with neuroretinal detachment and uveitis. The therapy with Trametinib and Dabrafenib was stopped immediately after eye examination showing chorioretinopathy, nevertheless uveitis appeared thereafter first on the left eye and after 2 weeks on the right eye. Dabrafenib was finally stopped and the patient reported of an improvement of the ocular complications until the therapy with both chemotherapeutics was started again. One year after the appearance of ocular side effects the visual acuity is stable and no inflammatory progress is detected.

We observed 14 patients receiving Trametinib and Dabrafenib due to metastatic melanoma for one and a half year. One patient developed anterior uveitis, one showed neuroretinal detachment and one showed both uveitis and neuroretinal detachment.

The largest study reporting ocular side effects under therapy with MEK-inhibitors with 32 patients treated with Binimetinib was conducted by Urner-Bloch et al. finding only mild and self-limiting retinopathy [10]. A few authors published brief reports of patients receiving Trametinib and Dabrafenib and developing severe panuveitis [11] and multiple serous retinal detachment [12].

In conclusion, patients treated with MEK-inhibitors, especially Trametinib and Dabrafenib, should undergo consecutive eye examinations as in some patients’ symptoms do not exist or can be mild [9]. Treatment doses should be revaluated based on the severity of ocular side effects. Further studies are needed to evaluate the potential dose-related effect of ocular side events.

## Abbreviations

MAPK: Mitogen-activated protein kinase
MEK: Mitogen-activated protein kinase
